# A Long-Term Time Series of *Dinophysis acuminata* Blooms and Associated Shellfish Toxin Contamination in Port Underwood, Marlborough Sounds, New Zealand

**DOI:** 10.3390/toxins11020074

**Published:** 2019-02-01

**Authors:** Lincoln A. Mackenzie

**Affiliations:** Cawthron Institute, 98 Halifax Street, Nelson 7010, New Zealand; lincoln.mackenzie@cawthron.org.nz; Tel.: +64-3-548-2319

**Keywords:** *Dinophysis acuminata*, dinophysistoxins, pectenotoxins, Port Underwood, New Zealand

## Abstract

Blooms of the dinoflagellate *Dinophysis acuminata* occur every year in an important mussel cultivation area in Port Underwood, Marlborough Sounds, New Zealand. Annual maximum cell numbers range from 1500–75,000 cells L^−1^ and over 25 years of weekly monitoring the *D. acuminata* bloom has never failed to exhibit peaks in abundance at some time between spring and autumn. During winter (June–August) the dinoflagellate is often undetectable, or at low levels (≤100 cells L^−1^), and the risk of diarrhetic shellfish poisoning (DSP)-toxin contamination over this period is negligible. Bloom occurrence may be coupled to the abundance of *D. acuminata* prey (*Mesodinium* sp.) but the mechanism by which it maintains its long-term residence in this hydrologically dynamic environment is unknown. The toxin profile of *D. acuminata* is dominated by pectenotoxin-2 (PTX-2) and dinophysistoxin-1 (DTX-1), but the cellular toxin content is low. It is rare that free DTX-1 is detected in mussels as this is invariably exclusively present as fatty acid-esters. In only five out of >2500 mussel samples over 16 years have the levels of total DTX-1 marginally exceeded the regulated level of 0.16 mg kg^−1^. It is also rare that free PTX-2 is detected in mussels, as it is generally only present in its hydrolysed non-toxic PTX-2 seco acid form. The *D. acuminata* alert level of 1000 cells L^−1^ is often exceeded without DTX-1 residues increasing appreciably, and this level is considered too conservative.

## 1. Introduction

Since the phenomenon was first identified [1,2], diarrhetic shellfish poisoning (DSP) caused by various species of planktonic dinoflagellate in the genus *Dinophysis* (and some benthic *Prorocentrum* spp.) has become a significant quality assurance issue for shellfish aquaculture worldwide. *Dinophysis* spp. produce a suite of lipophilic polyether secondary metabolites within the okadaic acid (OA, DTX-1, DTX-2) and pectenotoxin (PTX-1–PTX-11) families. These toxins are internationally regulated at a maximum permissible level of <0.16 mg kg^−1^ [3] and there is a high level of awareness in the New Zealand industry that the avoidance of the harvesting of shellfish contaminated with these toxin residues is essential.

**Figure 1:**
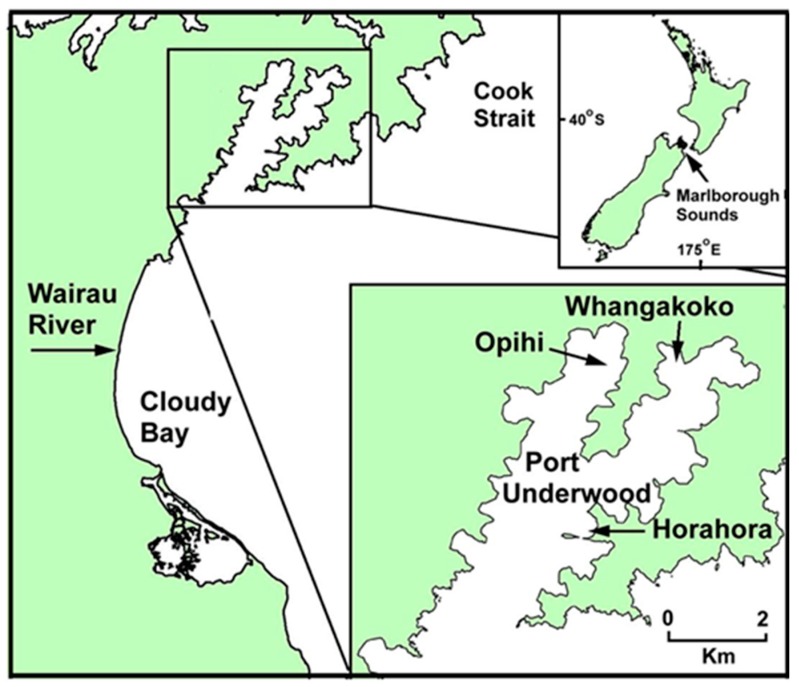
The geographic location of Port Underwood in the Marlborough Sounds region on the north-east coast of the South Island, New Zealand. The location of routine phytoplankton and shellfish monitoring sites (Opihi, Whangakoko, Horahora) in the inlet are indicated.

Port Underwood is a 24 km^2^ inlet on the north-east coast of the South Island, New Zealand (Figure 1), and is regarded as one of the most productive Greenshell^TM^ mussel (*Perna canaliculus*) growing regions of the Marlborough Sounds. The high productivity of the inlet (approximately 8000 tonnes of mussels per annum) is attributed to the fertilising effects of local wind-induced upwelling and its proximity to the Wairau River out-welling plume. Since weekly toxic phytoplankton and marine biotoxin monitoring began in Port Underwood in the early 1990s, it has become apparent that the inlet has a resident population of *Dinophysis acuminata* that blooms for short periods every year between spring (September–October) and autumn (March–April).

The cellular toxin content of the major pectenotoxin and okadaic acid group toxins in *Dinophysis acuta* and *Dinophysis acuminata* from various locations around the South Island coast of New Zealand has been described [4]. *D. acuminata* cells sampled from Port Underwood at various times showed low levels of okadaic acid (trace–0.4 pg/cell) and dinophysistoxin-1 (DTX-1) (0.1–0.5 pg cell^−1^) but much higher levels of pectenotoxin-2 (PTX-2) (2.4–16.9 pg cell^−1^). The cellular content of OA and its esters in *D. acuta* was 33 times higher and for PTX-2 and PTX-11, 8–42 times higher, respectively, than in *D. acuminata*. Only about 6% of total PTX-2 was present as the PTX-2 seco acid in *D. acuminata* cells. The toxin content of *D. acuminata* cells from Port Underwood was significantly lower than that of the same species from elsewhere in the South Island (Akaroa Harbour). DTX-1 was not detected in any *D. acuta* cells and DTX-2 was not detected in either species. A similarly low toxin content (0.01–1.8 pg OA + DTX-1 cell^−1^) has been observed in *D. acuminata* cells from the north-western Atlantic [5].

Pectenotoxins are the predominant polyether macrolides found in the *Dinophysis* species throughout the world [6]. At least 11 PTX analogues have been described, with different degrees of toxicity as assayed by various methodologies [7]. Pectenotoxin 2 seco acid (PTX-2sa) was first isolated and described in mussels from the Marlborough Sounds, New Zealand and Ireland [8]. Subsequently, it was shown [9] that PTX-2sa was the product of the rapid enzymatic hydrolysis of PTX-2 within shellfish tissues and the esterase responsible for this conversion was isolated and characterised from the mussel hepatopancreas [10]. This enzymatic conversion severs the PTX-2 lactone ring essential for the biological activity of the molecule and thus, PTX-2sa loses its toxicity [7]. In the early 2000s, as the extent of PTX-2sa occurrence in New Zealand shellfish became apparent [11], an evaluation of the risk of this compound to consumers was undertaken [12]. This study concluded that available evidence suggested that PTX-2sa was harmless. The New Zealand shellfish regulatory authority (New Zealand Ministry for Primary Industries) sets a limit of 0.16 mg kg^−1^ okadaic acid equivalents that must not be exceeded in the edible portion of the shellfish. Okadaic acid group toxins (e.g., DTX-1) and pectenotoxins (e.g., PTX-2) are considered additive and, above this level, shellfish harvesting is prohibited. Pectenotoxin-seco acid analogues (e.g., PTX-2sa) are not considered a hazard and there is no non-permissible level.

The data presented here, accumulated over 25 years of weekly monitoring of phytoplankton and shellfish, provide a unique perspective on the magnitude of the DSP-toxin contamination problem in Port Underwood. Additional data from the occasional opportunistic sampling of the water column within the inlet illuminate some aspects of *D. acuminata* ecology and the environmental circumstances accompanying dinophysis-toxin contamination events.

## 2. Results and Discussion

### 2.1. *Dinophysis acuminata* Morphology

**Figure 2:**
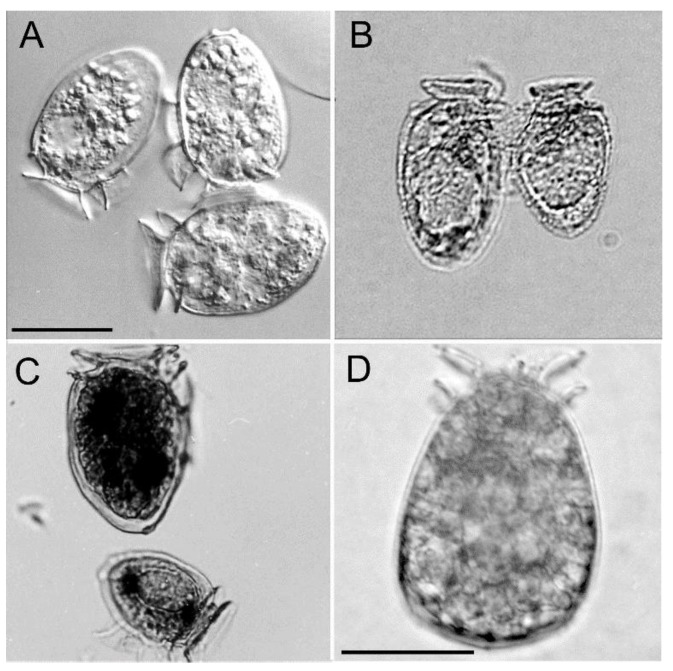
Specimens of *Dinophysis acuminata* from Port Underwood. (**A**) Live *D. acuminata* vegetative cells. (**B**) Conjugation of anisogamous gametes. (**C**) Large and small cell forms. (**D**) Dorsal view of a large, red, swollen cell. The scale bar (20 µm) in (**A**) also applies to (**B**,**C**). The scale bar in (**D**) is also 20 µm.

The vegetative cells of Port Underwood *D. acuminata* were on average (*n* = 10) 41.3 ± 2.7 µm (cell length) by 27.6 ± 1.6 µm (cell width) and, in terms of cell size and general morphology (Figure 2A), were consistent with descriptions of *D. acuminata* from elsewhere in the world [5]. A smaller morphotype (31 × 21 µm) was also commonly observed (Figure 2C), occasionally fusing with the larger morphotype. These fusing cells represent mating anisogamous gamete pairs and have been observed in several other *Dinophysis* species [13,14,15].

A third morphotype was comprised of large, swollen (50 × 33 µm), deeply red-pigmented cells that were most commonly observed during the early phases of bloom development (Figure 2D) It was believed these cells had recently fed on *Mesodinium* sp. prey [16] and that they played an important role in the subsequent rapid increase in cell numbers seen during subsequent blooms. Over the 25 years of monitoring in Port Underwood, larger cells of *D. acuta* have been observed on a few occasions. In October–November 2009, cell numbers of *D. acuta* briefly reached a maximum of 800 cells L^−1^ but no reportable associated toxicity in mussels occurred and this species has not played a significant role in DSP-toxin contamination events in this location to date.

### 2.2. Frequency of *Dinophysis acuminata* Blooms

**Figure 3:**
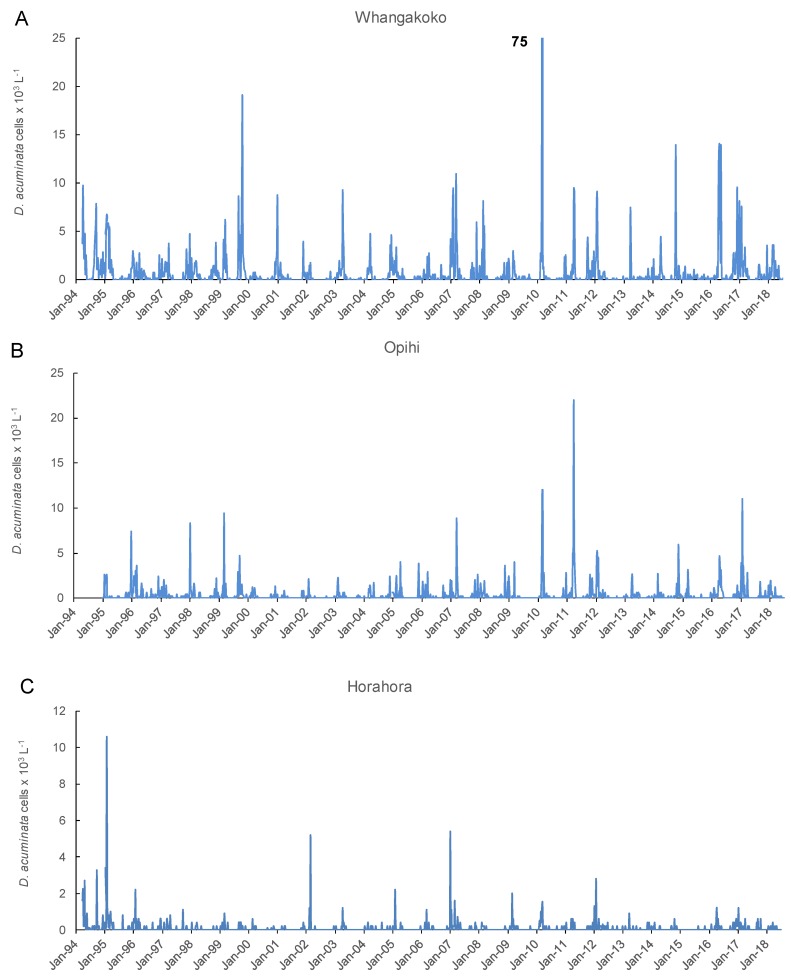
*Dinophysis acuminata* cells numbers (×10^3^ L^−1^) in 15 m water column tube samples collected weekly at three monitoring sites in Port Underwood 1994–2018. (**A**) Whangakoko Bay; (**B**) Opihi Bay; (**C**) Horahora Bay.

**Figure 4:**
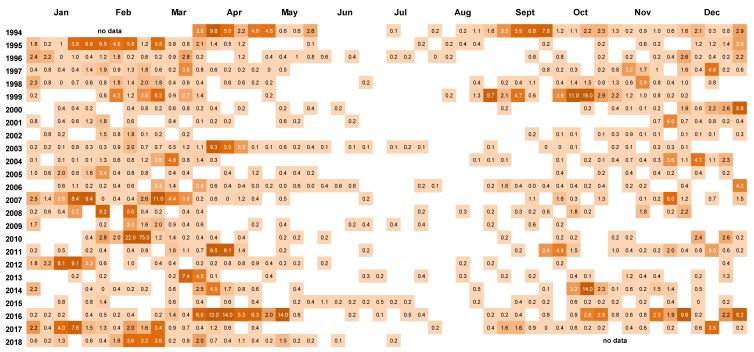
Cell counts (cells × 10^3^ L^−1^) of *Dinophysis*. *acuminata* in weekly 15 m tube samples from Whangakoko Bay, Port Underwood, March 1994–July 2018. Blank spaces indicate cell numbers <10^2^ cells L^−1^.

**Figure 5:**
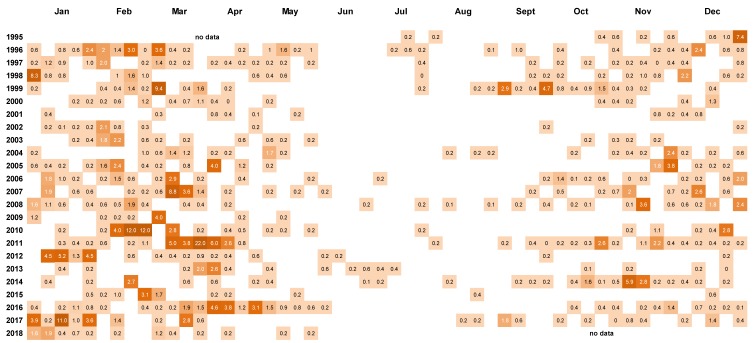
Cell counts (cells × 10^3^ L^−1^) of *Dinophysis acuminata* in weekly 15 m tube samples from Opihi Bay, Port Underwood, 1995–2018. Blank spaces indicate cell numbers <10^2^ cells L^−1^.

Peaks in *D. acuminata* abundance occurred at some time between spring and autumn every year from 1994 to 2018 (Figure 3, Figure 4 and Figure 5). Periods of relative abundance generally occurred at the same time at the three sampling sites and cell numbers were usually higher at the more inland sites (Whangakoko, Opihi) than at the Horahora site further towards the mouth of the inlet. The highest annual peak cell numbers usually occurred at the Whangakoko site and ranged from 2800–75,000 cells L^−1^. Mid-winter (June–August) was the period when cells were most likely to be absent from the plankton (Figure 4 and Figure 5), though even then there were a few occasions when cell numbers exceeded 1000 cell L^−1^.

### 2.3. Spatial and Temporal Distribution of Blooms

**Figure 6:**
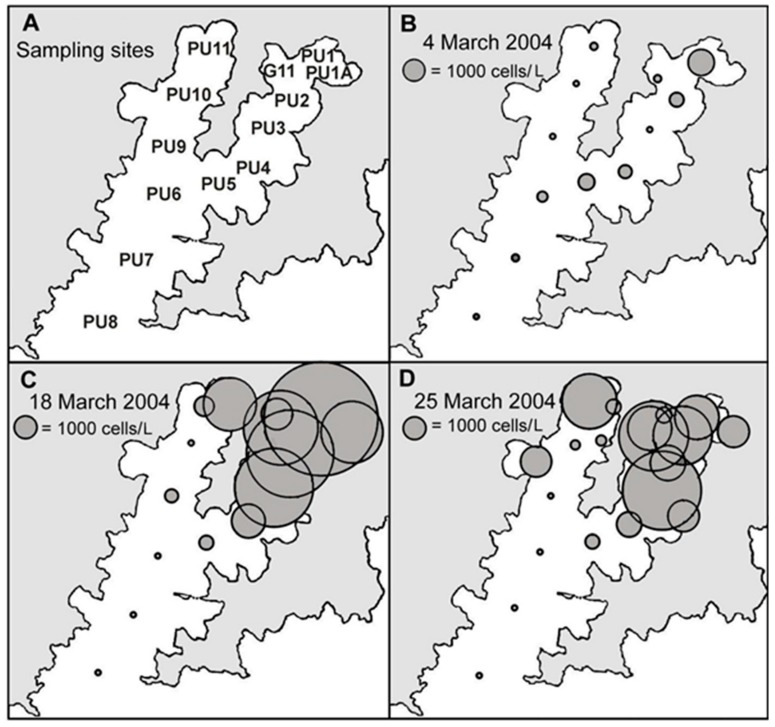
The distribution of cells in 15 m water column tube samples during a bloom of *Dinophysis acuminata* in Port Underwood, March 2004. (**A**) Sample site designations. (**B**) 4 March 2004; (**C**) 18 March 2004; (**D**) 25 March 2004.

**Figure 7:**
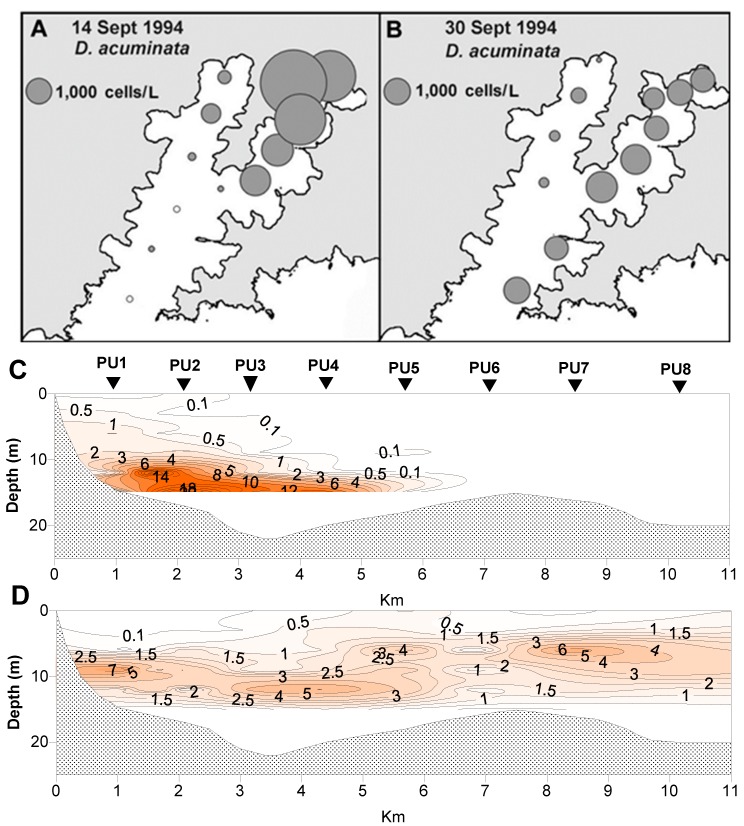
Spatial and vertical distribution of *Dinophysis acuminata* during a bloom between 14 Sept and 30 Sept 1994. (**A**,**B**) Spatial distribution of cells in 15 m tube samples. (**C**,**D**) The vertical distribution of cells along the Whangakoko Arm transect. The values are in cells × 10^3^ L^−1^. The site designations (PU1–PU8) are the same as shown in Figure 6.

**Figure 8:**
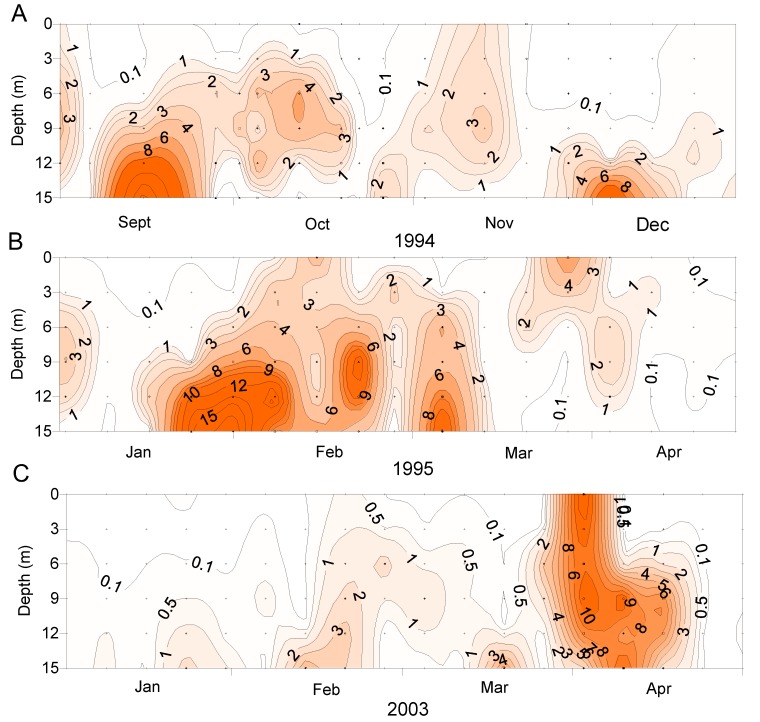
The vertical distribution of *Dinophysis acuminata* in the water column at Whangakoko, Port Underwood, at various times. (**A**) Sept–Dec 1995; (**B**) Jan–Apr 1995; (**C**) Jan–Apr 2003.

*D. acuminata* blooms developed in the inland reaches of the two major arms of the inlet (Figure 6 and Figure 7), becoming more widely dispersed (spatially and with depth) as the blooms matured (Figure 7 and Figure 8). In early March 2004 (Figure 6), as the bloom was in its early stages, *D. acuminata* cell numbers were highest at 12–15 m depth, in association with a phytoplankton community dominated by diatoms (*Chaetoceros* spp.) and the phototrophic ciliate *Mesodinium* sp. A significant proportion of the *D. acuminata* populations at these depths (14% at 12 m and 23% at 15 m) were comprised of the large, swollen, red-pigmented cells described previously (Figure 2). These observations are consistent with a conceptual model of the *Dinophysis* spp. growth strategy [17,18] and it is believed that the appearance of these large cells signals the imminent rapid development of a bloom. In cultures of *D. acuminata* [16], cell division rates of up to 0.95 day^−1^ have been observed when light and *Mesodinium* sp. prey abundance were non-limiting.

**Figure 9:**
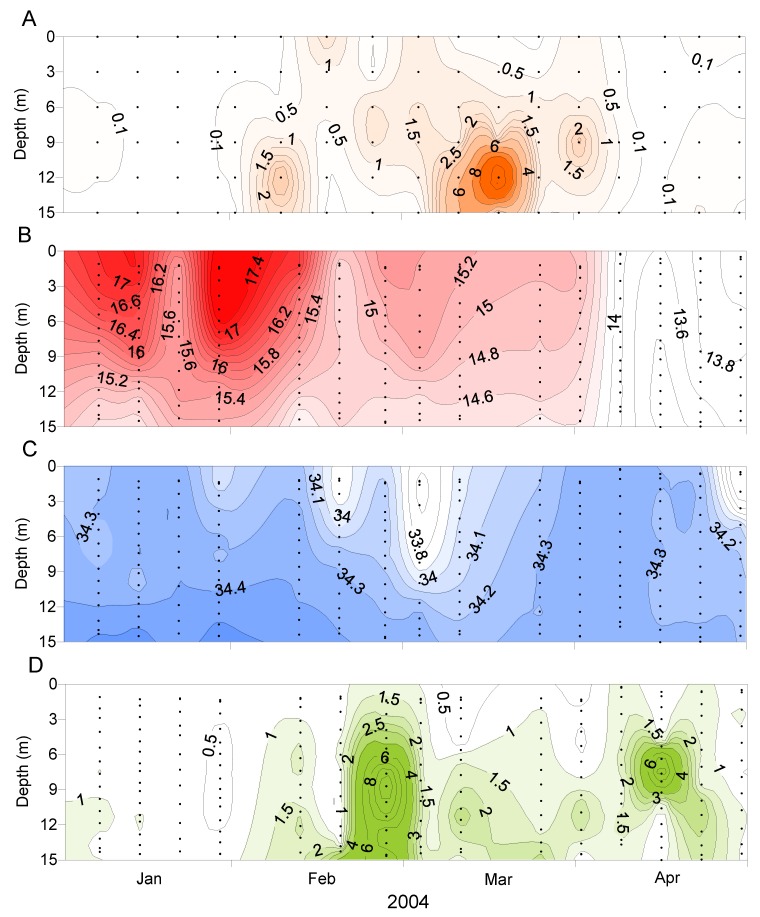
The progression of a *Dinophysis acuminata* bloom relative to the water column conditions at the Whangakoko site, January-April 2004. (**A**) *D. acuminata* cell numbers (cells × 10^3^ L^−1^). (**B**) Temperature (°C). (**C**) Salinity. (**D**) Chlorophyll a concentration (µg L^−1^).

*D. acuminata* blooms occurred at times when the water column was strongly stratified (due to salinity and temperature) but outside of the main phytoplankton bloom periods represented by high chlorophyll a concentrations (Figure 9). It has been observed elsewhere that *Dinophysis* populations tend to increase when the water column is thermally stratified [19,20].

### 2.4. Toxins Originating from *D. acuminata* in Cultivated Mussels (*Perna canaliculus*)

Data from analyses using an LC-MS/MS multi-residue method for the determination of lipophilic algal toxins in shellfish [21,22] became available from early 2002.

**Figure 10:**
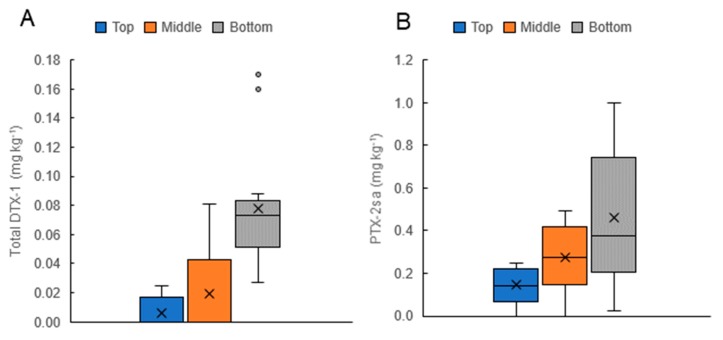
Summary of the depth distribution of total dinophysistoxin-1 (DTX-1) (**A**) and pectenotoxin-2sa (PTX-2sa) (**B**) Concentrations in Greenshell^TM^ mussels on 14 occasions when samples were collected from the vertical mussel culture ropes, near the surface (Top), in the middle of the rope (approx. 6 m), and at the bottom of the rope (approx. 12 m). Error bars show the range of values. Horizontal lines are the medium values and crosses indicate the means. Dots show values considered outliers by this analysis (in this case, values at or above the regulatory level of 0.16 mg kg^−1^).

On 14 occasions during *D. acuminata* blooms, the sampling of mussels at three depths on the vertical culture lines (near the surface, at 6 m, and at 12 m) was carried out (Figure 10). The distribution of the toxins in these shellfish (higher concentrations of PTX-2sa and total DTX-1 deeper in the water column) paralleled the depth distribution of *D. acuminata* cells (Figure 7, Figure 8 and Figure 9).

**Figure 11:**
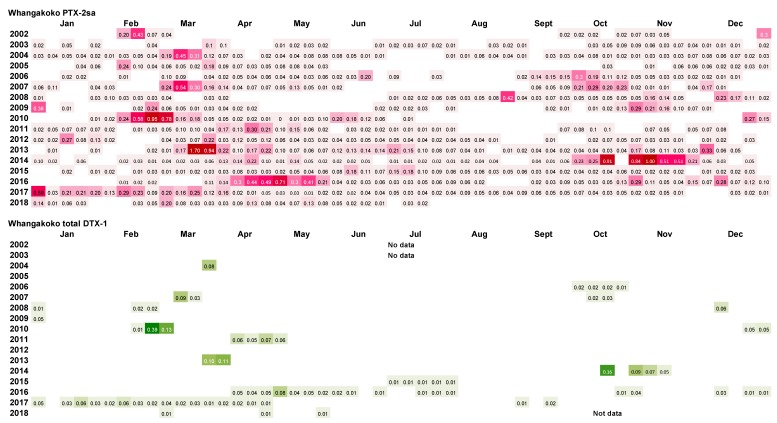
*Dinophysis acuminata* secondary metabolites (PTX-2sa and total DTX-1) in Greenshell^TM^ mussels from Whangakoko, 2002–2018. Blank spaces indicate levels below the limit of reporting (<0.01 mg kg^−1^ of PTX-2sa and total DTX-1).

**Figure 12:**
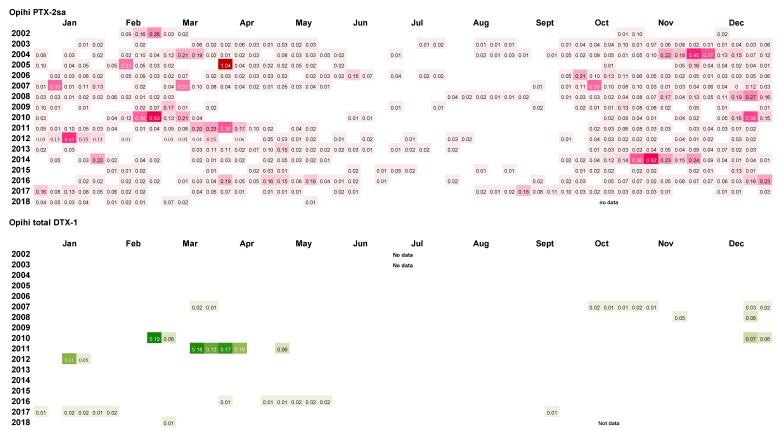
*Dinophysis acuminata* secondary metabolites (PTX-2sa and total DTX-1) in Greenshell^TM^ mussels from Opihi, 2002–2018. Blank spaces indicate levels below the limit of reporting (<0.01 mg kg^−1^ of PTX-2sa and total DTX-1).

A summary of the results of the weekly analysis of mussel tissues from the Whangakoko and Opihi monitoring sites (Figure 11 and Figure 12) shows that PTX-2sa was present above the reporting level (0.01 mg kg^−1^) in the majority (70.6% and 54.5%, respectively) of samples analysed at both sites. At the Horahora site, 22.6% of samples had PTX-2sa above the reporting level. The maximum concentrations of PTX-2sa observed at Whangakoko, Opihi, and Horahora were 1.7 mg kg^−1^ (March 2013), 1.4 mg kg^−1^ (April 2005), and 0.24 mg kg^−1^ (October 2009), respectively. At Whangakoko, parent PTX-2 was only above the reporting level in 27 out of 860 samples analysed (3.1%), with a maximum concentration of 0.039 mg kg^−1^ in April 2016. At Opihi, only 1.1% of samples contained reportable levels of PTX-2, with a maximum concentration of 0.025 mg kg^−1^ (January 2012). At Horahora, free PTX-2 was not detected. It is likely that the concentrations of PTX-2sa were, in fact, significantly higher than those reported here, since PTX-2sa-esters were not included in these analyses. According to Torgeston et al. [23], >80% of PTX-2sa in mussels (*Mytilus edulis*) may be in the form of fatty acid esters, with 16:0 and 14:0 predominant. Likewise, Blanco et al. [24] found that the concentrations of PTX-2sa and the palmytol ester of PTX-2sa were approximately equivalent in the digestive gland of the surf clam *Mesodesma donacium*. They also found that PTX-2 and PTX-2sa were more rapidly eliminated from the digestive gland than PTX-2sa esters.

**Figure 13:**
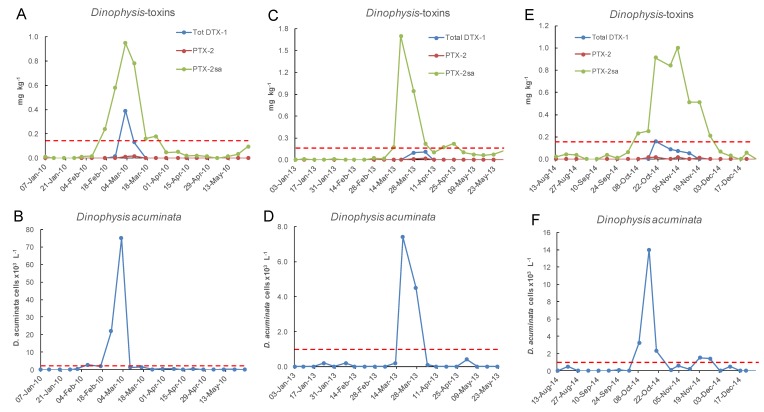
Three occasions between January 2002 and June 2018 at the Whangakoko sampling site when total DTX-1 approached or exceeded 0.1 mg kg^−1^, in relation to the cell numbers of *Dinophysis acuminata* in the water column. (**A**,**B**) 7 Jan–26 May 2010; (**C**,**D**) 3 Jan–23 May 2013; (**E**,**F**) 17 Aug–28 Dec 2014. The dashed lines indicate the maximum permitted level of 0.16 mg kg^−1^ total DTX-1 in shellfish and the action level of 1 × 10^3^ cells L^−1^ of *Dinophysis acuminata.*

**Figure 14:**
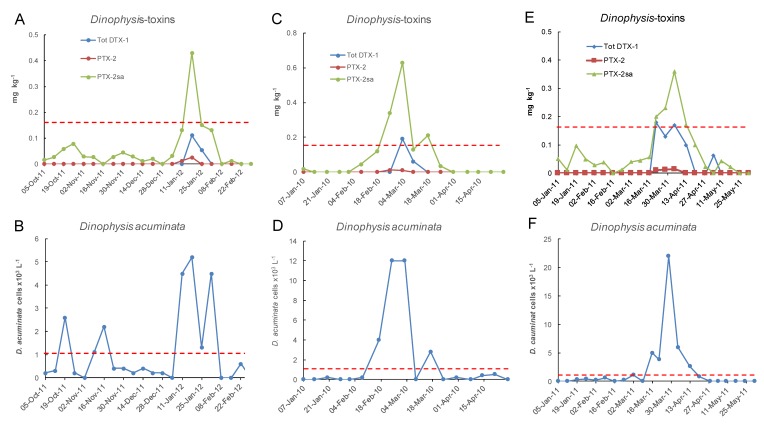
Three occasions between January 2007 and June 2018 at the Opihi sampling site when total DTX-1 approached or exceeded 0.1 mg/kg in relation to cell numbers of *Dinophysis acuminata* in the water column. (**A**,**B**) 8 Oct 2011 to 25 Feb 2012; (**C**,**D**) 7 Jan 2010 to 28 Apr 2010; (**E**,**F**) 5 Jan 2011 to 31 May 2011. The dashed lines indicate the maximum permitted level of 0.16 mg kg^−1^ total DTX-1 in shellfish and the action level of 1 × 10^3^ cells L^−1^ of *Dinophysis acuminata*.

Only a small proportion of samples between 2002 and 2018 had concentrations of total DTX-1 above the reporting level (Figure 10 and Figure 11). DTX-1 was the only okadaic acid group toxin identified in Port Underwood mussels between 2002 and 2018 and was usually only detectable after the alkaline hydrolysis of fatty acid esters within the extracts. Free DTX-1 was only detected on a few occasions. At Whangakoko and Opihi, 8.5% and 4.2% of samples, respectively, had total DTX-1 above the reporting level (Figure 11 and Figure 12). At Horahora, between February 2002 and July 2018 there was only one single report of DTX-1 in mussels (0.05 mg/kg) on 9 March 2010. The highest concentration of DTX-1 observed was 0.39 mg kg^−1^ at Whangakoko on 3 March 2010. Between October 2003 (when the alkaline hydrolysis procedure was introduced) and July 2018, 2307 samples were screened for the total DTX-1. Of these, only 11 samples (0.4%) had a total DTX-1 above 0.1 mg kg^−1^ and only 5 samples (0.2%) had concentrations at or above the regulatory level of 0.16 mg kg^−1^ (Figure 13 and Figure 14). The cell numbers of *D. acuminata* on all of these occasions exceeded 5000 cells L^−1^ and were accompanied by peak concentrations of PTX-2sa (0.3–1.0 mg kg^−1^). Abal et al. [25] have recently shown through the oral dosing of mice, that the toxicity equivalency factors (TEFs) between okadaic acid and dinophysistoxins −1 and 2 are ranked as OA = 1, DTX-1 = 1.5, and DTX-2 = 0.3. Applying a 1.5 factor to these, DTX-1 concentration data only slightly increased the number of samples exceeding 0.16 mg kg^−1^ from five to nine per 2307 samples analysed (0.2–0.3%).

## 3. Conclusions

Port Underwood has a resident population of *D. acuminata* that, over 24 years of continuous weekly monitoring, has never failed to bloom to some degree, at some time, from early spring to late autumn. Blooms developed in near-bottom waters in the most inland reaches of the inlet. It is believed that the abundance of *Mesodinium* sp. prey at depth may be an important precursor of the blooms. *Mesodinium* sp. is common in the inlet but cells are poorly preserved in Lugol’s iodine and numbers were not recorded during routine monitoring, so no definitive data exists to demonstrate the relationship between populations of prey and predators. *D. acuminata* numbers rarely exceed 10,000 cells L^−1^ but low numbers of cells are present in the water column throughout much of the year. Cultivated mussels show evidence of this in the high proportion of samples that show low concentrations of (non-regulated) PTX-2sa in their flesh. The DTX-1 content of *D. acuminata* cells is low and only a small proportion of mussel samples show evidence of DTX-1 accumulation (primarily as DTX-1 fatty acid-esters) above the limit of reporting. Over 16 years of weekly LC-MS analysis, only a small proportion of mussel samples (0.2%) achieved or marginally exceeded the regulated level of 0.16 mg kg^−1^. In every case when this level was reached, *D. acuminata* cell numbers exceeded 10,000 cells L^−1^. Cell numbers of up to 5000 cells L^−1^ did not result in toxicity exceeding the regulatory limit, and the current action level of 1000 cells L^−1^ could be reviewed. It has been shown in cultured and natural populations of *Dinophysis* spp. [26,27] that the toxin quota can vary by an order of magnitude during the growing season, with the maximum cell content being exhibited during the stationary phase. It is possible that the toxin quota also varies temporally in the Port Underwood *D. acuminata* but the long-term data set presented here clearly shows that, given the inherent low toxicity of this population, any natural fluctuations in toxin quota are unlikely to be of any practical significance with respect to the toxin content of cultivated mussels. Increasing concentrations of PTX-2sa are a good indicator of the imminence of DTX-1 contamination and the addition of PTX-2sa ester quantification to the routine monitoring protocol would likely increase the sensitivity of this indicator.

## 4. Materials and Methods

Sea-water samples were collected weekly at three sites (Whangakoko, Opihi, and Horahora) in Port Underwood (Figure 1) with a tube sampler that provided a ≤15-metre depth-integrated sample of the water column. On some occasions, samples from selected depths were also collected with a van Dorn sampler. Surveys of water column properties (temperature, salinity, and chlorophyll a fluorescence) were carried out using a Chelsea Instruments “Aquapack” CTD instrument (West Molesly, Surrey, UK). Phytoplankton identification and counts were carried out on Lugol’s iodine preserved samples, after the settling of 10 mL aliquots in Utermöhl chambers and examination under an inverted microscope.

Samples of Greenshell^TM^ mussels (*Perna canaliculus*) were collected weekly from mussel culture long-lines at the same sites as the phytoplankton samples. Routinely, samples were collected from a depth of 6 m, although on some occasions during significant bloom events, additional samples were also collected from near the surface (top) and from a depth of 12 m (bottom). Phytoplankton and shellfish samples were couriered to the Cawthron phytoplankton and biotoxin laboratories and the results of the analyses were available within 24 hours of receipt of the samples.

Prior to 2002, diarrhetic shellfish poisoning (DSP) toxicity of shellfish samples was analysed using the standard mouse bioassay [2], after solvent extraction using the revised method of Hannah et al. [28]. However, because of the poor quantification and unreliability of the results of these tests, none of these data are included in this analysis.

From 2002, all mussel samples were analysed using the multi-residue LC-MS/MS method for lipophilic algal toxins developed and validated by McNabb et al. [21,22]. Shellfish tissue homogenates (from a minimum of 12 fresh specimens) were blended with 90% aqueous methanol and the centrifuged extract was cleaned-up with a hexane wash. LC-MS/MS was used for the quantitative analysis with reversed phase gradient elution (Luna C18 5µm 150 × 2mm column; acidic buffer), electrospray ionisation (positive and negative ion switching), and multiple reaction monitoring (MRM). The MRM channels were monitored in windows that covered the elution of the compounds of interest (precursor > daughter): okadaic acid −ve 803.5 > 255.0, +ve 827.5 > 723.4; DTX-1 −ve 817.5 > 255.2, +ve 841.5 > 723.4; PTX-2 +ve 876.6 > 823.2; PTX-2 seco acid +ve 894.5 > 823.5. Okadaic acid and PTX-2 were quantified with reference to certified reference materials (CRM-OA-d, CRM PTX2-b) from the National Research Council, Canada. DTX-1 and PTX-2 seco acid were also calibrated with reference to these standards after the application of relative response factors. Ester forms of dinophysistoxins (in this case, exclusively DTX-1 esters) were detected as the parent toxin following alkaline hydrolysis of the methanolic extract [29] and were reported as the total DTX-1.

Until 2016, the lower limits of reporting of PTX-2, PTX-2 seco acid, and total DTX-1, the only lipophilic toxin residues of any significance in these shellfish, were <0.01, <0.01, and <0.05 mg kg^−1^, respectively. From 2016 onwards, the limit of reporting of the total DTX-1 was also reduced to <0.01 mg kg^−1^.
